# Supplementary material for: Inhibition of Glucose Uptake Blocks Proliferation but Not Cytotoxic Activity of NK Cells
Source: Cells. 2022 Nov 3;11(21):3489. doi: 10.3390/cells11213489 (PMC9655024; doi:10.3390/cells11213489)
Supplement: Supplementary file 1 [file cells-11-03489-s001.zip › cells-1976150-supplementary.pdf]

cells

Supplementary Materials

Inhibition of Glucose Uptake Blocks Proliferation but Not Cytotoxic Activity of NK Cells

Lea Katharina Picard [1], Elisabeth Littwitz-Salomon [2], Herbert Waldmann [3,4] and Carsten Watzl [1,*]

[1] Department for Immunology, Leibniz Research Centre for Working Environment and Human Factors at TU Dortmund (IfADo), D-44139 Dortmund, Germany
[2] Institute for Virology, University Hospital Essen, D-45147 Essen, Germany
[3] Max Planck Institute of Molecular Physiology, D-44227 Dortmund, Germany
[4] Chemical Biology, Faculty of Chemistry, Technical University Dortmund, D-44227 Dortmund, Germany
* Correspondence: watzl@ifado.de

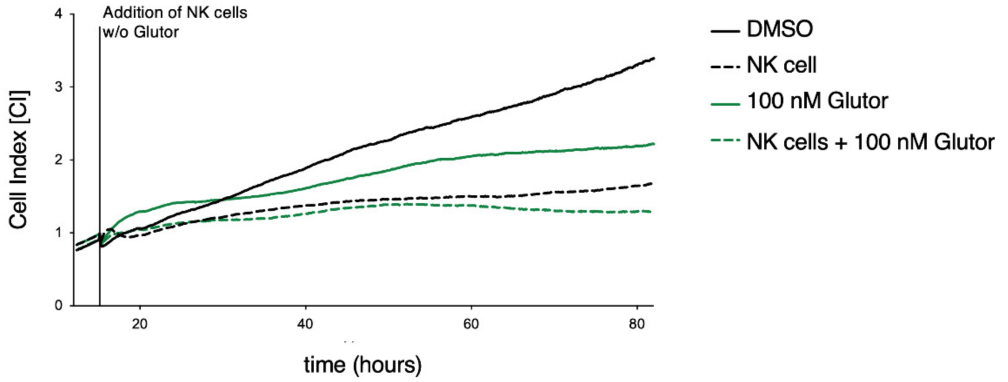

Figure S1. Impedance-based analysis of MCF7 cells. Representative graph of the impedance-based analysis of MCF7 cells in the presence or absence of 0.185x104 NK cells, 100 nM Glutor or 0.185x104 NK cells + 100 nM Glutor.

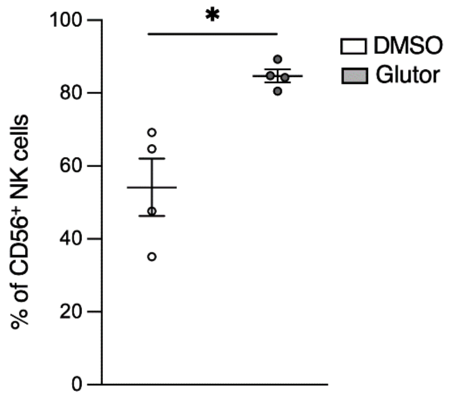

Figure S2. Enhanced GLUT-1 expression on NK cells after long-term treatment with Glutor. Expression level of GLUT-1 was analyzed on day 21 of long-term treated NK cells (100 nM Glutor or 0.1% DMSO). n=4. Data were pooled from two independent experiments each experiment was performed with two donors. Statistics: paired t-test. Statistics: paired t-test. significant differences are indicated by asterisk *P ≤ 0.05, **P ≤ 0.01, ***P ≤ 0.001, ****P ≤ 0.0001.

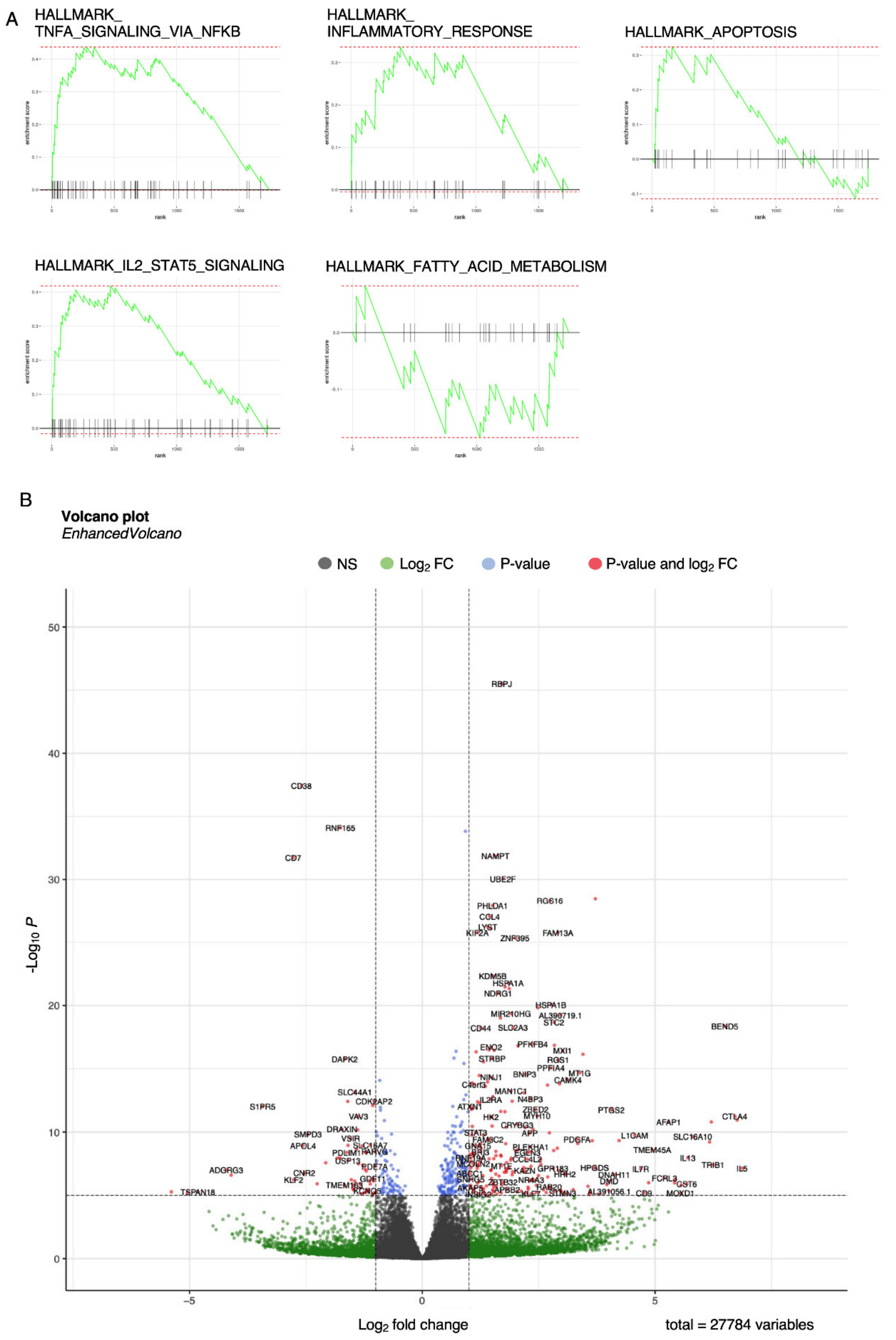

Figure S3. RNA sequencing results. A) GSEA of long-term treated NK cells. Hallmark apoptosis, hallmark fatty acid metabolism, hallmark inflammatory response, hallmark Il2 STAT5 signaling, hallmark TNF-α signaling via NFκb n=6. B) Volcano plot displaying differences in relative gene expression after long-term treatment with Glutor. Plot represents the –log10 of the p-value against log2 fold change. Grey dots: non-significant; green dots: log2 fold change significant; blue dots: p-value significant; red dots: p-value and log2 fold change significant. n=6

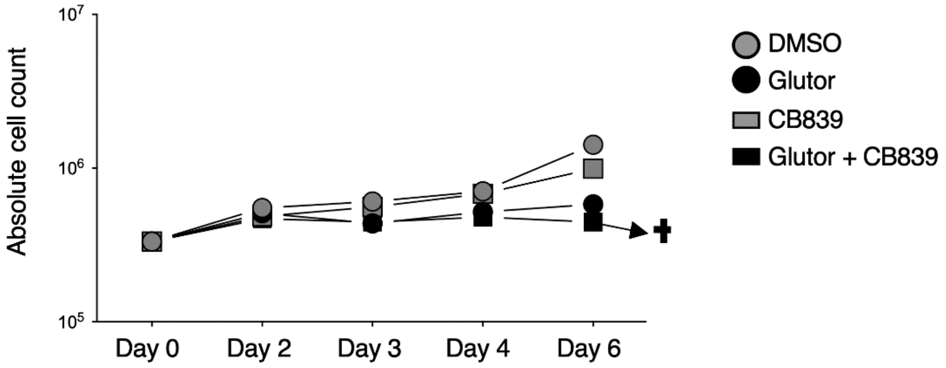

Figure S4. Inhibition of Glutaminase and glucose transporters results in cell death of NK cells. Proliferation of Glutor-treated (100 nM), Glutaminase inhibitor (CB839)-treated (0,5 μM) or CB839 + Glutor-treated NK cells in comparison to control (DMSO)-treated NK cells. Absolute cell count was calculated at indicated time points. n=4. At day 6 no living cells could be recovered from the Glutor+CB839 treated sample (indicated by †). Data were pooled from three independent experiments each experiment was performed with one or two donors.
